# Upregulation of p27 cyclin-dependent kinase inhibitor and a C-terminus truncated form of p27 contributes to G1 phase arrest

**DOI:** 10.1038/srep27829

**Published:** 2016-06-10

**Authors:** Takayuki Satoh, Daisuke Kaida

**Affiliations:** 1 Frontier Research Core for Life Sciences, University of Toyama, 2630 Sugitani, Toyama 930-0194, Japan; 2 Graduate School of Medicine and Pharmaceutical Sciences, University of Toyama, 2630 Sugitani, Toyama 930-0194, Japan

## Abstract

Potent anti-cancer compounds FR901464 and its methyl-ketal derivative spliceostatin A (SSA) inhibit cell cycle progression at G1 and G2/M phases. These compounds bind to the spliceosome and inhibit the splicing reaction. However, the molecular mechanism underlying G1 arrest after SSA treatment remains unknown. In this study, we found that ~90% of SSA-treated cells arrested at G1 phase after cell cycle synchronization. SSA treatment caused upregulation of the p27 cyclin-dependent kinase inhibitor both at mRNA and protein levels. In addition to p27, we observed expression of p27*, a C-terminal truncated form of p27 that is translated from *CDKN1B* (p27) pre-mRNA accumulated after splicing inhibition. Overexpression of p27 or p27* inhibited the exit from G1 phase after a double thymidine block. Conversely, knocking down of p27 by siRNA partially suppressed the G1 phase arrest caused by SSA treatment. There results suggest that G1 arrest in SSA-treated cells is caused, at least in part, by upregulation of p27 and p27*.

Cell cycle progression is tightly regulated by the cyclin family proteins, cyclin-dependent kinases (CDKs), and CDK inhibitors. A major player in G1 progression is the p27 CDK inhibitor^1^,^2^. p27 binds to the cyclin E-CDK2 complex, which plays important roles in the transition into S phase, and inhibits the function of the complex to control cell cycle progression in G1 phase^3^,^4^,^5^,^6^,^7^. Consistent with the molecular function of p27, its protein level is high at G0 and early G1 phases and declines during G1 phase^1^,^8^. Thus, for accurate cell cycle progression, decrease in p27 protein level at the right timing is required^1^. Indeed, overexpression of p27 causes G1 phase arrest^5^,^7^. The protein level of p27 is mainly controlled at the post-transcriptional level, both at translation and degradation stages. The most characterized regulation mechanism is the degradation of p27 by the ubiquitin-proteasome pathway^9^,^10^,^11^. Ubiquitination of p27 by the SCF^Skp2^ E3 ubiquitin ligase is triggered by phosphorylation of the threonine 187 residue of p27^12^,^13^.

Splicing of pre-mRNA is one of the essential steps to maintain the integrity of the transcriptome^14^,^15^. The splicing reaction is carried out by the spliceosome, a macromolecular ribonucleoprotein complex that consists of five major components: U1, U2, U4, U5, and U6 small ribonucleoprotein particles (snRNPs). These snRNPs bind to pre-mRNA to perform the splicing reaction. The potent splicing inhibitor spliceostatin A (SSA), which is a methyl-ketal derivative of FR901464, binds to U2 snRNP and inhibits the splicing reaction *in vivo* and *in vitro*^16^,^17^. Previous studies showed that FR901464 also exhibits anti-cancer activity^17^. SSA and FR901464 cause cell cycle arrest at G1 and G2/M phases, and this cell cycle arrest is thought to be the mechanism underlying the anti-cancer activity^16^,^18^.

SSA and FR901464 also induce expression of a C-terminus truncated form of p27, designated as p27*, which is translated from pre-mRNA^16^,^18^. *CDKN1B*, which encodes p27 protein, consists of three exons and two introns. Translation starts from the start codon in exon 1 and terminates at the stop codon in exon 2 to produce p27 protein. Splicing inhibition causes accumulation of intron 1, and translation from the start codon in exon 1 to the first in-frame stop codon in intron 1 leads to production of p27*, which inhibits Cdk2 activity^16^. Production of p27* may be a likely explanation for the G1 arrest after SSA treatment; however, no direct evidence has shown that p27* inhibits cell cycle progression. In this study, we investigated the molecular mechanism underlying G1 arrest after SSA treatment and the potential involvement of p27 and p27* in SSA-mediated cell cycle arrest.

## Results

### SSA treatment causes cell cycle arrest at G1 phase

**Figure 1:**
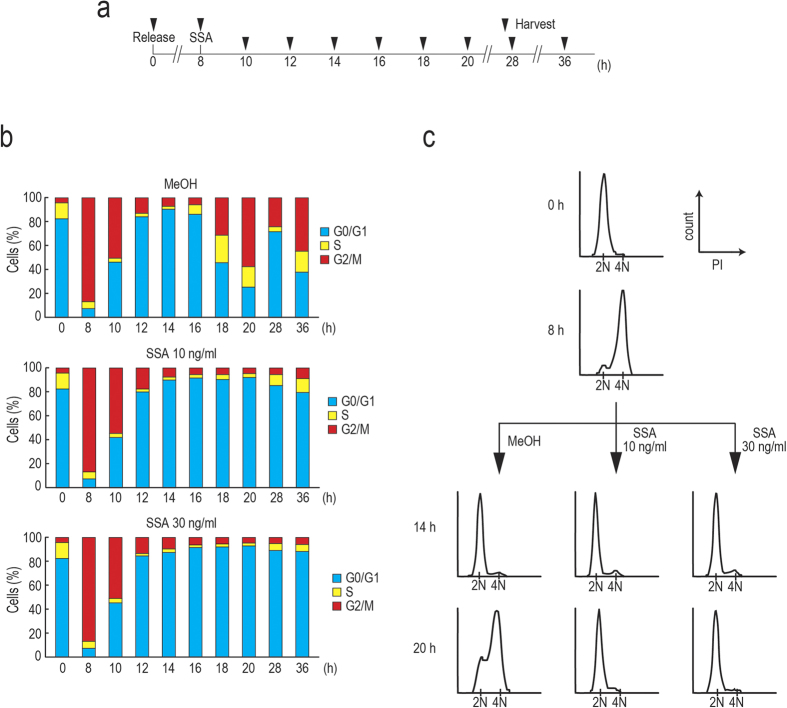
SSA treatment causes G1 arrest. (**a,b**) Eight hours after release from a double thymidine block, synchronized HeLa S3 cells were treated with MeOH or 10 ng/ml or 30 ng/ml of SSA. The cells were harvested at the indicated time points and cell cycle progression was analyzed using cytometry (n = 3). (**c**) Representative histograms from (**b**).

Previous reports showed that SSA treatment inhibits cell cycle progression of non-synchronized cells at G1 and G2/M phases^16^,^18^. However, it is difficult to distinguish whether cell cycle progression is arrested at G1 and G2/M phases or if cell cycle progression is only delayed at these phases using non-synchronized cells. To investigate if SSA causes G1 arrest, we examined the effect of SSA treatment on cell cycle progression of synchronized cells. We treated HeLa S3 cells with thymidine to synchronize the cell cycle at the end of G1 phase. Eight hours after the release from thymidine block, we treated the cells with MeOH or SSA and cell cycle progression was monitored using a cytometer (Fig. 1a). As expected, most of the cells were in M phase at the beginning of SSA treatment (8 h) and most of both MeOH- and SSA-treated cells were in G1 phase at 12–16 h (Fig. 1b,c). MeOH-treated cells entered S phase again and transited to G2/M phase at 20 h, whereas ~90% of SSA-treated cells remained in G1 phase at 20 h. Most of the SSA-treated cells continued to remain in G1 phase at 36 h, suggesting that SSA has potent cell cycle arrest activity at G1 phase.

### SSA treatment causes upregulation of p27 and production of p27*

**Figure 2:**
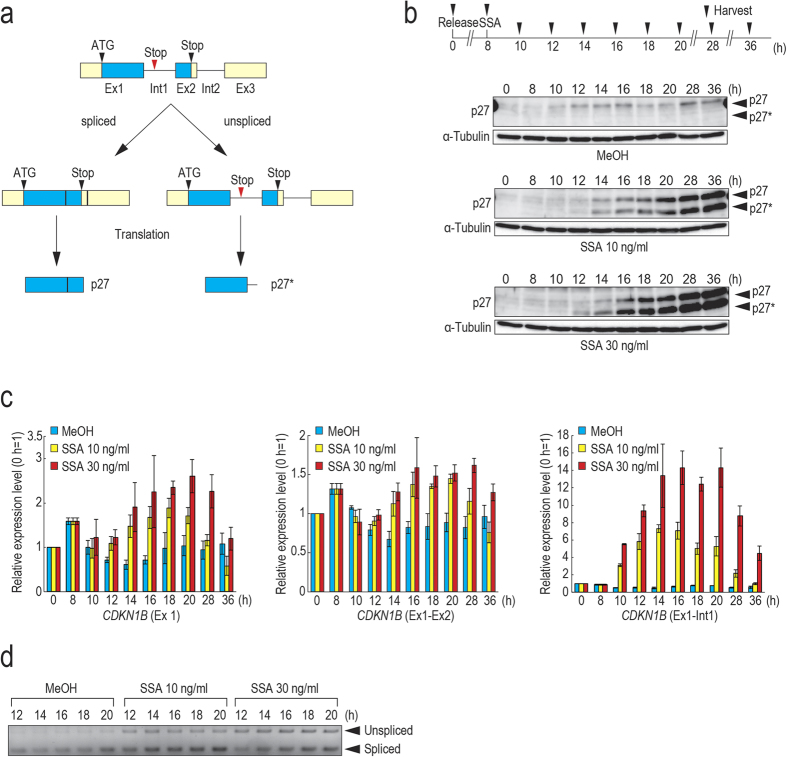
SSA treatment upregulates p27 at mRNA and protein levels. (**a**) Gene structure of CDKN1B and a schema of production of p27 and p27*. Cyan boxes, light yellow boxes, and horizontal lines represent coding sequences in exons, untranslated regions of exons and introns, respectively. (**b**) Eight hours after release from a double thymidine block, synchronized HeLa S3 cells were treated with MeOH or 10 ng/ml or 30 ng/ml of SSA. The cells were harvested at the indicated time points and expression levels of p27 and p27* were analyzed by immunoblotting. Protein level of α-tubulin was analyzed as an internal control. (**c**) Total RNAs were prepared from the cells harvested at the indicated time points in (**b**) and analyzed by qRT-PCR to check the relative expression level of exon 1 (*CDKN1B* (Ex1)), the spliced form (*CDKN1B* (Ex1-Ex2)), and the unspliced form (*CDKN1B* (Ex1-Int1)) of the *CDKN1B* gene. Error bars indicate s.d. (n = 3). (**d**) Total RNAs were prepared as in (**c**) and analyzed by RT-PCR using primers annealing to p27 exon 1 and exon 2 to detect both spliced and unspliced forms.

A previous study showed that SSA treatment induces expression of p27*, a C-terminus truncated form of p27, and that p27* is one of the possible reasons underlying SSA-induced G1 arrest^16^. Thus, next we checked expression levels of p27 and p27* in SSA-treated cells. p27* is produced by translation from the start codon in exon 1 to the first in-frame stop codon in intron 1 (Fig. 2a)^16^. We synchronized the cell cycle using thymidine, and 8 h after release from a double thymidine block, the cells were treated with MeOH or SSA (Fig. 2b). The cells were harvested and expression levels of p27 and p27* were examined by immunoblotting. In MeOH-treated cells, p27 was expressed predominantly in G1 phase (Figs 1b and 2b, 12–16 h) and started declining before entering into S phase (Figs 1b and 2b, 18 h), consistent with previous reports^1^,^8^. No p27* was observed in MeOH-treated cells, whereas p27* was highly expressed in SSA-treated cells as expected. Interestingly, we also observed upregulation of p27 and the expression level continued to increase to high levels at 36 h (Fig. 2b).

To investigate whether the upregulation of p27 and production of p27* in SSA-treated cells are regulated at the mRNA level or protein level, we first evaluated the levels of exon 1 of *CDKN1B*, which encodes p27 protein, by qRT-PCR. The amount of *CDKN1B* mRNA increased after SSA treatment, suggesting that transcription of *CDKN1B* is activated by SSA treatment (Fig. 2c, *CDKN1B* (Ex1)). In addition, we measured the amount of spliced and unspliced forms of *CDKN1B.* As expected, substantial accumulation of the unspliced form was observed after SSA treatment (Fig. 2c, *CDKN1B* (Ex1-Int1)). Interestingly, a slight increase of the spliced form was also observed in SSA-treated cells (Fig. 2c, *CDKN1B* (Ex1-Ex2)), probably because splicing inhibition of *CDKN1B* by SSA treatment is partial and transcription activation counterbalances the decrease of the spliced form caused by splicing inhibition. A similar result was observed by RT-PCR (Fig. 2d). Taken together, these data show that SSA treatment causes splicing inhibition resulting in the production of p27*. In addition, SSA also upregulated p27 expression at both mRNA and protein levels. Because the increase in p27 protein level was more prominent than the level of the spliced form of p27 mRNA (Fig. 2b,c), p27 protein might be stabilized in SSA-treated cells. However, we cannot rule out the possibility that SSA induces p27 translation.

### Overexpression of p27 and p27* results in cell cycle arrest at G1 phase

**Figure 3:**
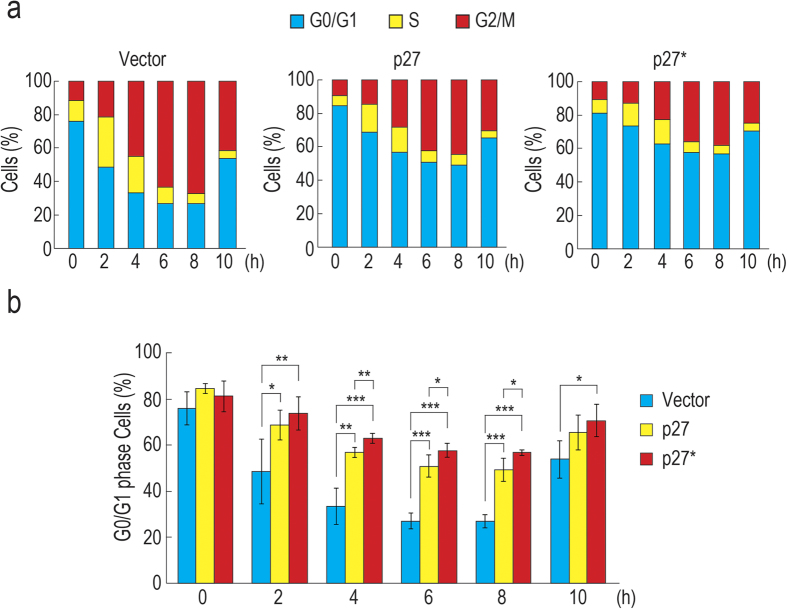
Overexpression of p27 and p27* inhibits cell cycle progression at G1 phase. (**a**) HeLa S3 cells were transfected with pcDNA3.1 (Vector), pcDNA3.1-FLAG-p27, or pcDNA3.1-FLAG-p27*. The transfectants were treated with thymidine to synchronize the cell cycle. After release from the thymidine block, the cells were harvested at the indicated time points and cell cycle progression was analyzed using a cytometer (n = 4). (**b**) Replot of data in (**a**). Error bars indicate s.d. (n = 4). Statistical significance was investigated by the unpaired two–tailed t-test (*P < 0.05; **P < 0.01; ***P < 0.001).

To investigate whether overexpression of p27 or p27* inhibits cell cycle progression at G1 phase, we subcloned DNA fragments encoding p27 or p27* into an expression vector. HeLa S3 cells were transfected with p27 or p27* plasmid and then treated with thymidine to synchronize the cell cycle. After release from a double thymidine block, cell cycle progression was assayed by a cytometer. The results showed that 67.2% of vector-transfected cells entered M phase at 8 h and then transited to G1 phase again at 10 h (Fig. 3a). We also found that 26.8% of the vector-transfected cells could not exit from G1 phase, presumably because of transfection stress. In contrast, 56.6% of p27*-overexpressing cells could not exit from G1 phase at 8 h, suggesting that overexpression of p27* causes cell cycle arrest at G1 phase. Although one-third of the overexpressing cells entered G2/M phase, this partial cell cycle arrest can be explained by transfection efficiency, which was ~70% (unpublished data, TS and DK). Overexpression of p27 also caused G1 arrest and 49.2% of the cells showed G1 arrest at 8 h, consistent with previous reports^5^,^7^. The proportion of G1 cells of vector-transfected cells was statistically significantly lower than that of p27- or p27*-overexpressing cells (Fig. 3b). These results suggest that overexpression of p27 and p27* causes cell cycle arrest at G1 phase.

### Knockdown of p27 and p27* suppresses the G1 arrest induced by SSA treatment

**Figure 4:**
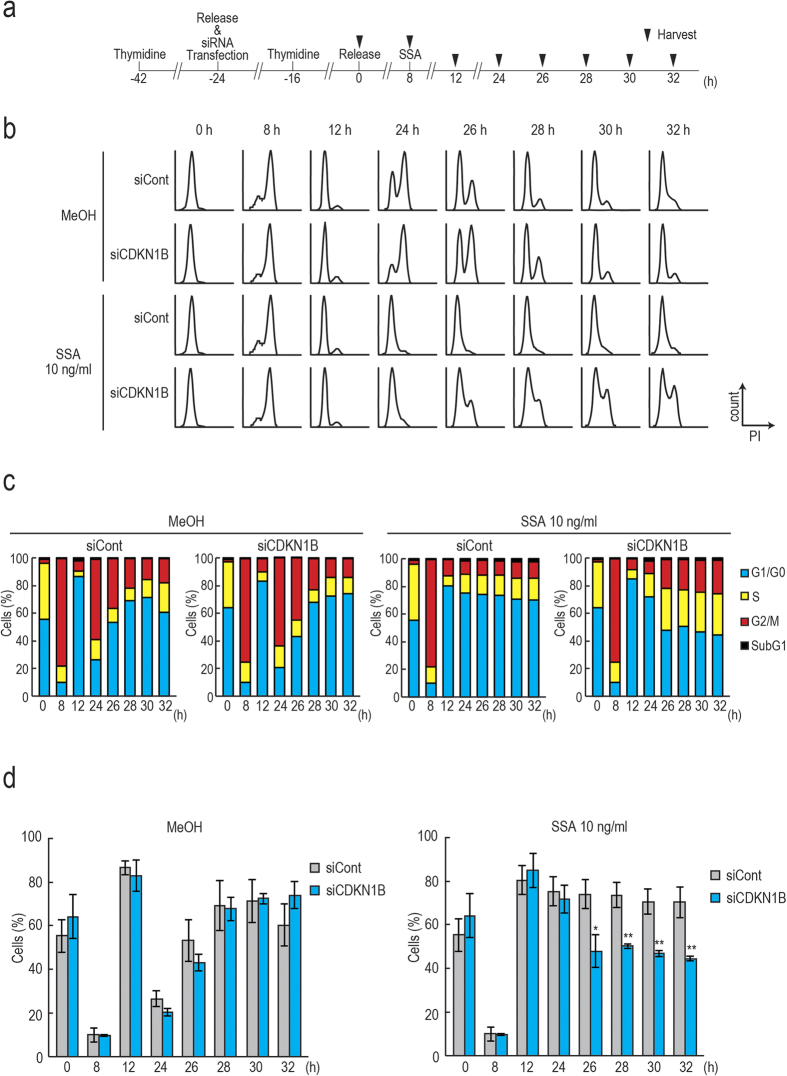
SSA-induced G1 arrest was suppressed by knockdown of p27. (**a**) Eighteen hours after treatment with thymidine, synchronized HeLa S3 cells were washed with fresh medium and transfected with p27 or control siRNA. Eight hours after the transfection, the cells were treated with thymidine again for 16 h. Eight hours after release from the second thymidine block, the cells were treated with SSA or MeOH and harvested at the indicated time points. (**b**) Representative histograms of the samples analyzed by the cytometer. (**c**) Proportion of the cells in each phase (n = 3). (**d**) Proportion of G1 arrested cells (n = 3). Error bars indicate s.d. (n = 3). Statistical significance was investigated by the unpaired two-tailed t-test (*P < 0.05; **P < 0.01; ***P < 0.001).

If overexpression of p27 and p27* contributes to the G1 arrest in SSA-treated cells, knockdown of p27 and p27* should suppress the G1 arrest caused by SSA treatment. To investigate the effect of p27 knockdown on cell cycle arrest in SSA-treated cells, we synchronized cells using thymidine, treated cells with *CDKN1B* siRNA and SSA, and then cell cycle progression was analyzed (Fig. 4a). The successful knockdown of p27 and p27* was confirmed by immunoblotting (Fig. S1a). The majority of MeOH-treated cells were in G2/M phase at 8 h, then in G1 phase at 12 h regardless of p27 knockdown (Fig. 4b–d). Most of the MeOH-treated cells were in G2/M phase again at 24 h and transited to G1 phase at 26–32 h, suggesting that cell cycle progression of MeOH-treated cells was not affected by p27 knockdown (Fig. 4b–d). Upon SSA treatment, control siRNA-transfected cells were arrested at G1 phase at 12–32 h (Fig. 4b–d). Cells treated with p27 siRNA and SSA were in G1 phase at 24 h, showing delay of exit from G1 phase (Fig. 4b–d). However, the cells exited from G1 phase slowly at 26–32 h, suggesting that p27 knockdown causes partial suppression of the G1 arrest by SSA treatment (Fig. 4b–d). These results suggest that p27 and/or p27* are required for the G1 arrest induced by SSA.

In addition, we treated cells with nocodazole to induce M phase arrest of cells after addition of SSA (Fig. S2a). If the cells were not arrested at G1 phase by SSA treatment, the cells should be arrested at M phase by nocodazole. This allows us to distinguish whether the cells were arrested at G1 phase by SSA or not. We confirmed efficient knockdown of p27 and p27^*^ by immunoblotting (Fig. S1b). Upon MeOH and nocodazole treatment, most cells arrested at M phase with or without siRNA treatment as expected (Fig. S2b). Upon SSA and nocodazole treatment, control siRNA-transfected cells showed an additional peak, which represents cells arrested at G1 phase, suggesting that SSA treatment induces G1 arrest (Fig. S2b, black arrow). Knockdown of p27 decreased the population of G1 arrested cells induced by SSA treatment (Fig. S2b, red arrow). We also calculated the proportion of the cells in each phase and found that p27 knockdown caused a decrease of G1 arrested cells caused by SSA treatment (Fig. S2c,d). These results also support the idea that overexpression of p27 and/or production of p27* contribute to the G1 arrest by SSA treatment.

Taken together, we propose that the cell cycle arrest at G1 phase in SSA-treated cells is caused, at least in part, by overexpression of p27 and production of p27*.

## Discussion

In this study, we found that ~90% of SSA-treated cells showed G1 arrest upon SSA treatment when the cells were mainly in M phase. As reported previously, SSA treatment causes cell cycle arrest at both G1 and G2/M phases^16^,^18^. Therefore, whether cell cycle progression is arrested at G1 or G2/M phase presumably depends on which phase of the cell cycle the cells are in when treated with SSA. We found that ~80% of SSA-treated cells showed G2/M arrest if the cells were treated with SSA when the cells were in G1/S (unpublished data, TS and DK).

To date, the molecular mechanism of G1 arrest after SSA treatment remains unknown. In this study, we confirmed that SSA treatment caused G1 arrest and upregulation of p27 and production of p27*. In addition, we found that overexpression of p27 or p27* resulted in cell cycle arrest at G1 phase and that knockdown of p27 suppressed the G1 arrest induced by SSA, suggesting that G1 arrest of SSA-treated cells is caused, at least in part, by overexpression of p27 and production of p27*.

One of the major functions of p27 is binding to the cyclin E-Cdk2 complex to inhibit CDK activity and G1 phase arrest^4^,^5^. Because p27*, like p27, inhibits CDK activity^16^, the upregulation of p27 and production of p27* observed in SSA-treated cells might cause cell cycle arrest through inhibition of cyclin E-Cdk2. Another possible mechanism of G1 phase arrest is inhibition of the cyclin D-Cdk4/6 complex, which is important, but not essential, for re-entry into the cell cycle^19^,^20^,^21^. In some contexts, p27 can inhibit cyclin D-Cdk4/6 activity and the inhibition might cause G1 arrest^22^. In addition, cyclin D-Cdk4/6 is important for cyclin E transcription^2^. Therefore, cell cycle arrest in SSA-treated cells might be caused through inactivation of the cyclin D-Cdk4/6 complex. In addition, we found that knockdown of p27 suppressed the G1 arrest induced by SSA, however the suppression was partial and we still observed a delay of exit from G1 phase. Therefore, some factors that are important for cell cycle progression at G1 phase, including cyclin D, cyclin E, Cdk2 and Cdk4/6, might be downregulated in SSA-treated cells. Although the detailed molecular mechanism of G1 arrest from SSA treatment is still unknown, our results revealed that overexpression of p27 and p27* inhibited cell cycle progression at G1 phase.

We also found that p27 expression was upregulated after SSA treatment both at mRNA and protein levels. However, p27 protein increased more considerably than the spliced form of p27 mRNA, suggesting that p27 protein level was mainly regulated at the post-transcriptional level in SSA-treated cells, for example at translation or degradation stages. The cyclin E-Cdk2 complex phosphorylates p27 at threonine 187, triggering p27 for ubiquitination and degradation by proteasome^9^,^10^. In SSA-treated cells, the protein levels of cyclin E-Cdk2 or proteasome components might decrease. Another possible mechanism of p27 upregulation is stabilization of p27 caused by p27* production. Because p27* does not have the phosphorylation site, p27* is thought to be more stable than p27^16^. As reported previously, p27* inhibits cyclin E-Cdk2, therefore overexpression of p27* might stabilize p27 through inhibition of cyclin E-Cdk2. Another possibility is downregulation of PTB, ELAVL1/4 or miR-221/222 by SSA treatment, because translation of p27 is regulated by these proteins and miRNAs^23^,^24^,^25^. Further studies are required to clarify the molecular mechanism underlying p27 upregulation in SSA-treated cells.

In this study, we found that ~90% of cells showed G1 arrest by SSA treatment after cell cycle synchronization and that SSA treatment caused upregulation of p27 and production of p27*. In addition, we revealed that overexpression of p27 and p27* caused cell cycle arrest at G1 phase and that knockdown of p27 suppressed the G1 arrest induced by SSA. Because SSA treatment causes cell cycle arrest, the splicing machinery can be a prospective target for novel anti-cancer drugs through cell cycle inhibition. Although the detailed molecular mechanism is still unknown, these findings serve to further our understanding of the mechanism of the interconnection between splicing and cell cycle arrest and development for cancer therapy.

## Methods

### Cell culture, synchronization, and reagents

HeLa S3 cells were cultured in Dulbecco’s modified Eagle’s medium (Wako, Osaka, Japan) containing 10% heat-inactivated fetal bovine serum (Life Technologies, Eugene, OR, USA) at 37 °C with 5% CO_2_. For cell cycle synchronization, cells were treated with 2 mM of thymidine (Wako) for 18 h. After treatment, the cells were washed twice with medium to release from a thymidine block and then cultured in fresh culture medium for 8 h. The cells were treated with 2 mM of thymidine again for 16 h and then washed with culture medium twice to release from a double thymidine block. Nocodazole was purchased from Sigma-Aldrich (St. Louis, MO, USA). SSA was a gift from Dr. M. Yoshida (RIKEN, Saitama, Japan).

### Antibodies and immunoblotting

Mouse monoclonal anti-α-tubulin (T6199) was purchased from Sigma-Aldrich. Rabbit polyclonal anti-p27 (N-20) (#sc-527) was purchased from Santa Cruz Biotechnology (Dallas, TX, USA). HRP-conjugated anti-mouse IgG and anti-rabbit IgG secondary antibodies were purchased from GE Healthcare (Little Chalfont, UK).

For immunoblotting, cells were suspended in lysis buffer (25 mM HEPES, pH 7.5, 150 mM NaCl, 2 mM MgCl_2_, 1 mM EGTA, 1 mM EGTA, 1% Nonidet P-40, 10% glycerol, cOmplete ULTRA tablets mini EDTA-free [Roche, Basel, Switzerland] and PhosSTOP [Roche]) and vortexed for 15 s. Cell lysates were subjected to immunoblotting with the indicated antibodies. Immune complexes were detected using the NOVEX ECL Chemiluminescent Substrate Reagent Kit (Life Technologies) on an ImageQuant LAS 4000mini (GE Healthcare).

### Cell cycle analysis

Cells were fixed in 70% ethanol, rinsed with phosphate-buffered saline, and stained with solution containing 20 μg/ml propidium iodide (Life Technologies), 0.05% Triton X-100, and 0.1 mg/ml RNase A (Life Technologies). Cell cycle progression was monitored by the image-based cytometer Tali (Life Technologies).

### siRNA transfection

Silencer select p27/CDKN1B siRNA (s2837 cat #4390824) was purchased from Life Technologies. siGENOME Control Pool Non-Targeting #2 (cat #D-001206-14-20) was purchased from GE Healthcare. siRNA transfection was performed using Lipofectamine RNAiMAX (Life Technologies) according to the manufacturer’s instructions.

### RNA purification, RT-PCR, and qRT-PCR

Total RNA was extracted from cells using the RNeasy mini kit with QIAshredder (Qiagen, Venlo, The Netherlands) following the manufacturer’s instructions. cDNA was prepared using Primescript II RTase (Takara, Otsu, Japan) and random primers. PCR reaction was performed using Takara ExTaq HS (Takara). Relative quantification analyses (qRT-PCR) were performed using a MX3000P system (Agilent, Santa Clara, CA, USA) using SYBR Green dye chemistry. The amount of 18S rRNA was measured as an internal control for qRT-PCR. All primers are listed in Table S1.

### Plasmids and transfection

To construct pcDNA3.1-FLAG, FLAG-tag F 5′-AGCTTATGGACTATAAGGACGATGATGACAAAGACTATAAGGACGATGATGACAAAGGTAC-3′ and FLAG-tag R 5′-CTTTGTCATCATCGTCCTTATAGTCTTTGTCATCATCGTCCTTATAGTCCATA-3′ were mixed and incubated at 95 °C for 1 min in TE buffer (10 mM Tris-HCl and 10 mM EDTA) and then slowly cooled down to 25 °C (−5 °C/min). The annealed dsDNAs were inserted into pcDNA3.1 (Life Technologies) between the *Hin*d III and *Kpn* I sites. The DNA fragment of p27 was amplified by PCR from a cDNA library using the primers p27 cloning F2 5′-CGCGGATCCATGTCAAACGTGCGAGTGTCT-3′ and p27 cloning R 5′-CGCGCGGCCGCTTACGTTTGACGTCTTCTGA-3′. The PCR product was digested with *Bam* HI and *Not* I and subcloned into pcDNA3.1-FLAG. The DNA fragment of p27* was amplified by PCR from genomic DNA of HeLa S3 cells using the primers p27 cloning F2 5′-CGCGGATCCATGTCAAACGTGCGAGTGTCT-3′ and p27* cloning R 5′-ATAGCGGCCGCTTAACACCCTCCAGCAGGC-3′. The PCR product was digested with *Bam* HI and *Not* I and subcloned into pcDNA3.1-FLAG. Plasmid transfection was performed using the Effectene Transfection Reagent (Qiagen) according to the manufacturer’s instruction.

## Additional Information

**How to cite this article**: Satoh, T. and Kaida, D. Upregulation of p27 cyclin-dependent kinase inhibitor and a C-terminus truncated form of p27 contributes to G1 phase arrest. *Sci. Rep.* **6**, 27829; doi: 10.1038/srep27829 (2016).

## Supplementary Material

Supplementary Information
