# Supplementary material for: Upregulation of p27 cyclin-dependent kinase inhibitor and a C-terminus truncated form of p27 contributes to G1 phase arrest
Source: Sci Rep. 2016 Jun 10;6:27829. doi: 10.1038/srep27829 (PMC4901259; doi:10.1038/srep27829)
Supplement: Supplementary Information [file srep27829-s1.pdf]

Upregulation of p27 cyclin-dependent kinase inhibitor and a C-terminus truncated form of p27 contributes to G1 phase arrest

Takayuki Satoh[1,2] and Daisuke Kaida[1,2]*

1 Frontier Research Core for Life Sciences, University of Toyama, 2630 Sugitani, Toyama 930-0194, Japan

2 Graduate School of Medicine and Pharmaceutical Sciences, University of Toyama, 2630 Sugitani, Toyama 930-0194, Japan

*To whom correspondence should be addressed.

Tel./Fax: +81-76-415-8848

E-mail: kaida@med.u-toyama.ac.jp

a

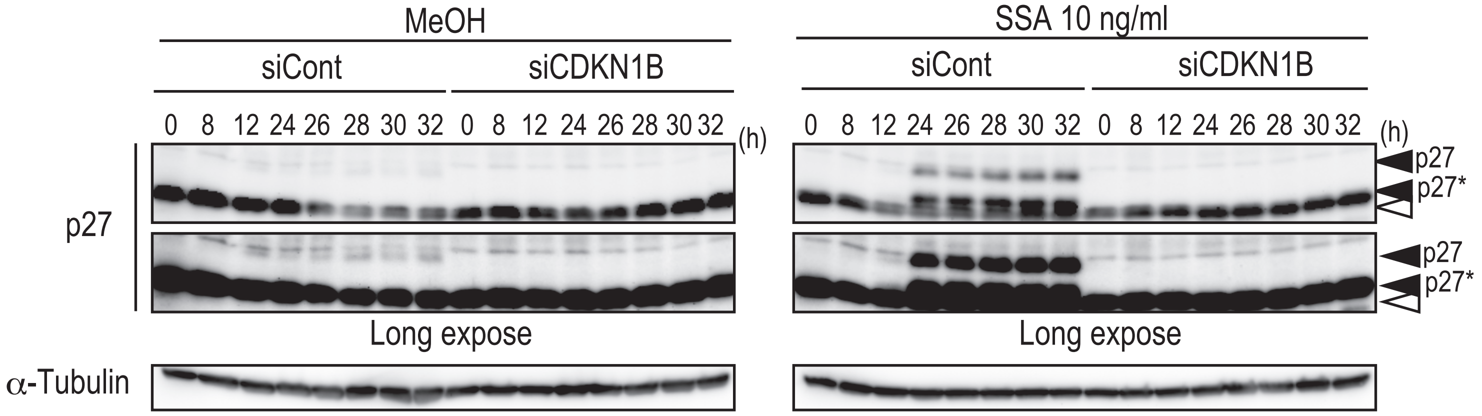

b

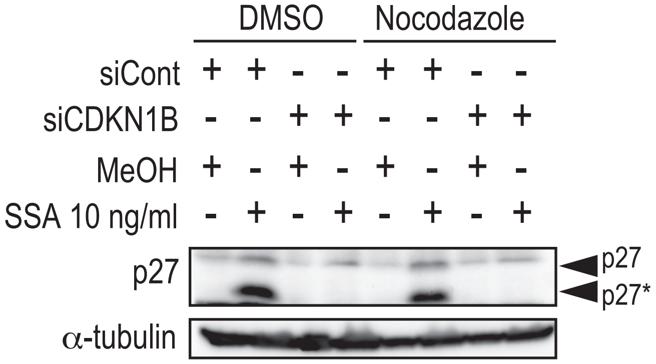

Figure S1. Successful knockdown of p27 and p27* proteins

We confirmed the successful knockdown of p27 and p27* proteins by immunoblotting using cells harvested at the same time points as in Fig. 4 and Fig. S2. White arrowheads indicate non-specific bands.

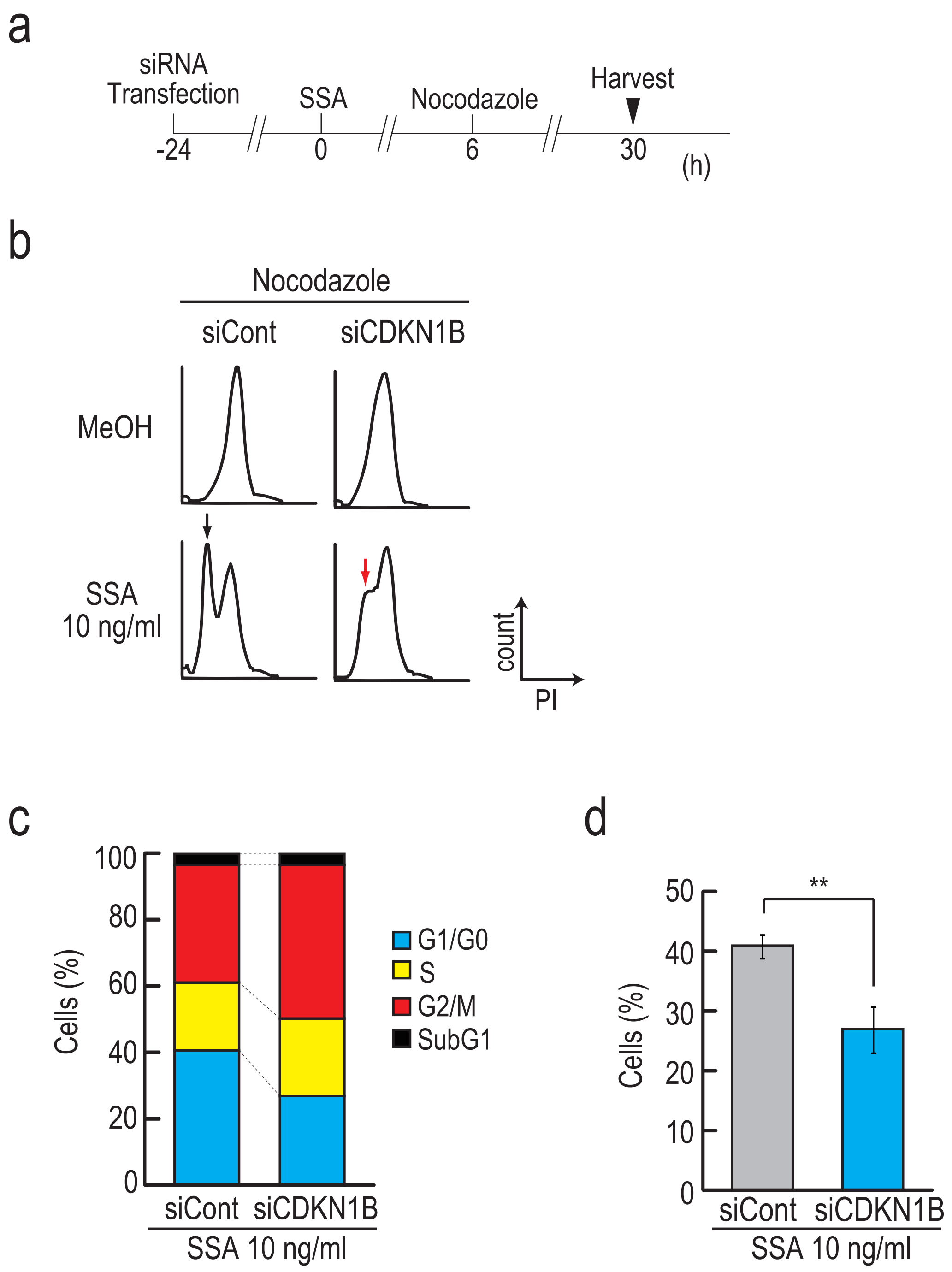

Figure S2. SSA-induced G1 arrest was suppressed by knockdown of p27

(a) HeLa S3 cells were transfected with p27 or control siRNA. After transfection, the cells were treated with 10 ng/mL of SSA and 100 ng/mL of nocodazole, and then harvested at the indicated time points. (b) Representative histograms of the samples analyzed by the cytometer.

(c) Proportion of the cells in each phase (n = 3). (d) Proportion of G1 arrested cells (n = 3). Error bars indicate s.d. (n = 3). Statistical significance was investigated by the unpaired two–tailed t-test (*P < 0.05; **P < 0.01; ***P < 0.001).

Supplementary Table 1. List of primer used in this study.

For qPCR	
Name	Sequence (5'-3')
hCDKN1B Ex1-2 qPCR F	TAAGGAAGCGACCTGCAACC
hCDKN1B Ex1-2 qPCR R	TTGACGTCTTCTGAGGCCAG
hCDKN1B Int1 qPCR R	AGCTCTCCCAAAGCTAAATCAGA
hCDKN1B Ex1 qPCR F	AGTGTCTAACGGGAGCCCTA
hCDKN1B Ex1 qPCR R	CCGGGTTAACTCTTCGTGGT
18S rRNA F	GTTGGTGGAGCGATTTGTCTGGTT
18S rRNA R	TATTGCTCAATCTCGGGTGGCTGA
For PCR	
Name	Sequence (5'-3')
hCDKN1B Ex1 for	CAAAGGTGCCTGCAAGGTG
hCDKN1B Ex2 rev	CACAGAACCGGCATTTGG
